# circASbase: A Comprehensive Database of Alternative Splicing Events in circRNAs

**DOI:** 10.1093/gpbjnl/qzaf121

**Published:** 2025-12-02

**Authors:** Lingxiao Zou (邹凌霄), Jian Zhao (赵健), Haojie Li (李豪杰), Chen Xu (许琛), Yulan Wang (汪玉兰), Xuejiang Guo (郭雪江), Xiaofeng Song (宋晓峰)

**Affiliations:** Department of Biomedical Engineering, Nanjing University of Aeronautics and Astronautics, Nanjing 211106, China; Department of Biomedical Engineering, Nanjing University of Aeronautics and Astronautics, Nanjing 211106, China; State Key Laboratory of Reproductive Medicine and Offspring Health, Department of Histology and Embryology, Nanjing Medical University, Nanjing 211166, China; Changzhou Medical Center, The Affiliated Changzhou Second People’s Hospital of Nanjing Medical University, Changzhou Second People’s Hospital, Nanjing Medical University, Changzhou 213000, China; Medical Research Center, Changzhou Maternal and Child Health Care Hospital, Changzhou Medical Center, Nanjing Medical University, Changzhou 213000, China; State Key Laboratory of Reproductive Medicine and Offspring Health, Department of Histology and Embryology, Nanjing Medical University, Nanjing 211166, China; Department of Biomedical Engineering, Nanjing University of Aeronautics and Astronautics, Nanjing 211106, China; State Key Laboratory of Reproductive Medicine and Offspring Health, Department of Histology and Embryology, Nanjing Medical University, Nanjing 211166, China; Department of Biomedical Engineering, Nanjing University of Aeronautics and Astronautics, Nanjing 211106, China

**Keywords:** Circular RNA, Alternative splicing, Full-length isoform, Coding potential, Database

## Abstract

Although extensive evidence has underscored the critical role of alternative splicing (AS) in generating mature circular RNA (circRNA) isoforms and augmenting their functional diversity, a significant gap remains in the availability of specialized databases housing circRNA AS events. To bridge this gap, we develop circASbase, a pioneering and comprehensive database that catalogs 452,129 AS events in 884,047 full-length circRNAs from 581 samples across 13 species, and provides rich annotations to facilitate understanding the splicing regulation of circRNA. Our findings reveal substantial differences between circRNAs and linear transcripts regarding the distribution and occurrence of AS events, highlighting the unique regulatory landscape of circRNAs. These special splicing events result in functional differences of circRNAs by affecting internal ribosome entry sites, *N*^6^-methyladenosine sites, open reading frames, protein features, microRNA targets, and more. In summary, circASbase not only meets the urgent need of the research community for data repositories, but also represents a significant advancement in our understanding of circRNA biology. With its user-friendly interfaces and web-based visualization tools, circASbase is poised to become an indispensable resource for researchers exploring the regulatory mechanisms and functional roles of AS events in circRNAs. This database will continuously drive new insights and discoveries in the field, setting the stage for further advancements in circRNA research. circASbase is freely available at http://reprod.njmu.edu.cn/cgi-bin/circASbase/.

## Graphical abstract

**Figure:**
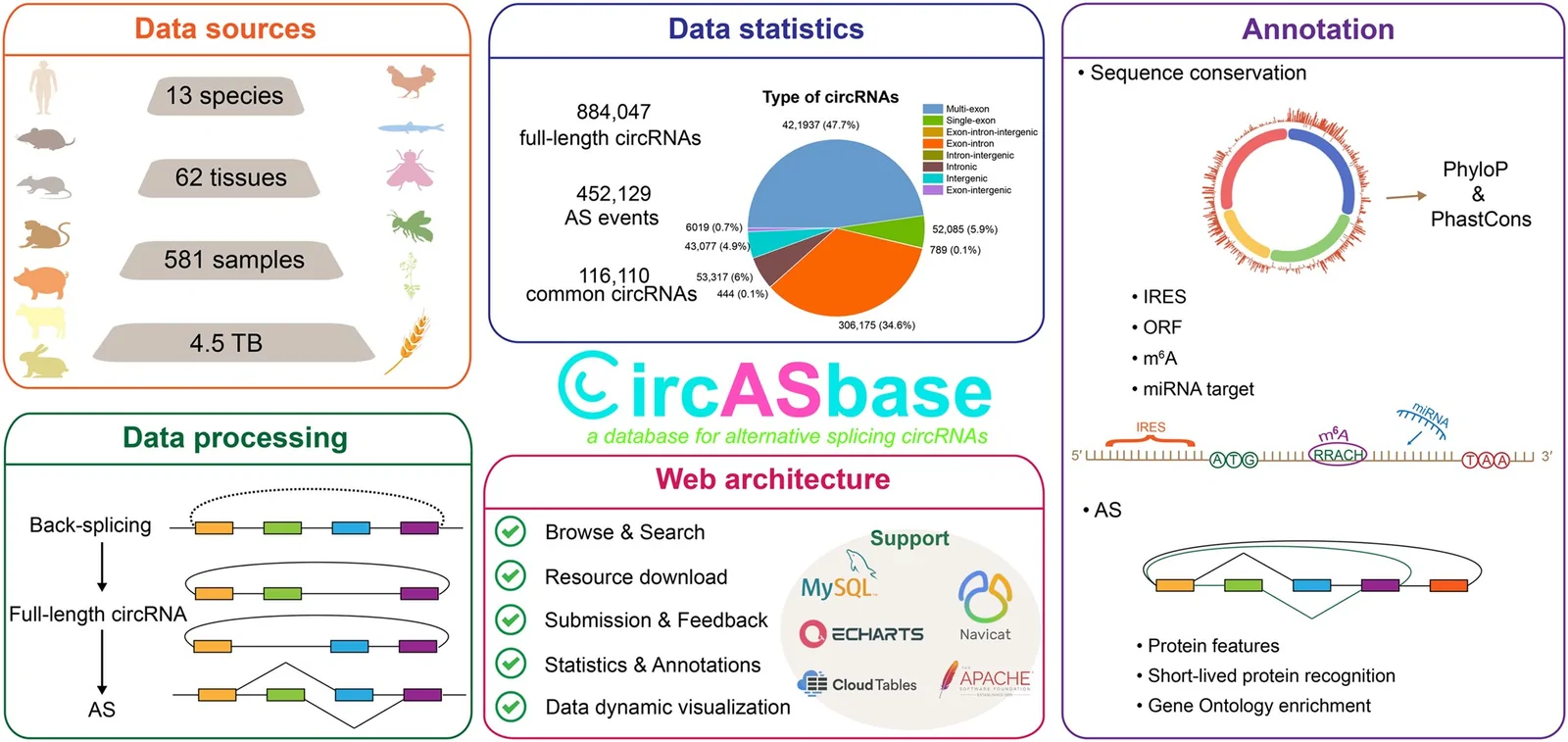

## Introduction

Circular RNAs (circRNAs) are a novel class of functional RNAs characterized by covalently closed-loop structures generated through back-splicing [1]. They are ubiquitously present in eukaryotes and exhibit tissue- and developmental stage-specific expression patterns [2–4]. Initially, circRNAs were thought to result from erroneous splicing, but Memczak et al. revealed that circRNAs act as sponges for microRNAs (miRNAs) [5–7]. Currently, circRNAs are recognized for their roles in diverse biological processes, including cell proliferation, migration, metastasis, and apoptosis [8]. Furthermore, growing evidence has revealed that dysfunction of circRNAs is closely associated with various diseases, particularly cancers [9,10].

Most circRNAs originate from precursor messenger RNAs (pre-mRNAs) and typically consist of multiple exons. While circRNAs are initially distinguished from their linear transcripts by back-splice junction (BSJ) sites, it is now known that multiple circRNAs can share the same BSJ via alternative splicing (AS) [11]. Furthermore, recent studies have indicated that circRNAs can be spliced distinctly from their linear counterparts [12,13]. Notably, several exons or introns have been found to be preferentially included in circRNAs through AS mechanisms [14]. Despite the discovery and investigation of a large number of circRNAs based on the recognition of BSJs, our understanding of AS events in circRNAs remains limited.

AS of messenger RNA (mRNA) and long non-coding RNA (lncRNA) has been extensively studied, and the association of RNA splicing regulation with various biological processes, such as sex determination, T-cell activation, and synapse development, has been well established [15–17]. Aberrant splicing has been associated with aging and various diseases, including cancers, inflammation, and metabolic disorders [18]. Similar to mRNA and lncRNA, the function of circRNA also relies primarily on its sequence, which determines miRNA-sponge activity, interactions with proteins, coding potential, *etc.* [19]. Dysregulation of AS can change circRNAs’ roles in biological processes, leading to dysfunction and disease [20,21].

Advances in RNA sequencing and analysis have led to the discovery of an increasing number of circRNAs. To deposit and annotate these circRNAs, several general and specialized databases have been developed, such as circBase [2], circRNADb [22], CircNet [23], CIRCpedia [24], Circbank [25], circAtlas [26], and CSCD [27]. Most of these databases use the BSJ as the unique identifier for circRNAs and generate their sequences by sequentially assembling exons located between the 5′ and 3′ ends of the BSJ. However, only a few databases, such as circAtlas [26], isoCirc [28], and PlantcircBase [29], provide full-length circRNA isoforms, which are essential for inferring AS events within circRNAs.

Recently, several methods have been proposed to assemble full-length circRNA isoforms. Zheng et al. introduced CIRI-full, which utilizes the BSJ and reverse overlap to reconstruct circular isoforms, based on a particular library preparation strategy [30]. Wu et al. developed CircAST to assemble alternatively spliced circRNA transcripts with RNase R-treated RNA sequencing (RNA-seq) data [31]. Xin et al. reported isoCirc for sequencing full-length circRNA isoforms using rolling amplification and long-read sequencing strategy [28]. Although thousands of AS events within the internal part of circRNAs have been identified, there is currently no specially designed database for depositing and annotating these splicing events. Here, we developed circASbase, a comprehensive database of AS events in circRNAs. circASbase provides systematic annotations for each splicing event, detailing its effect on circRNAs’ coding potential and regulatory features, such as internal ribosome entry sites (IRESs), *N*^6^-methyladenosine (m^6^A) sites, open reading frames (ORFs), protein features, and miRNA targets.

## Data collection and processing

**Figure 1:**
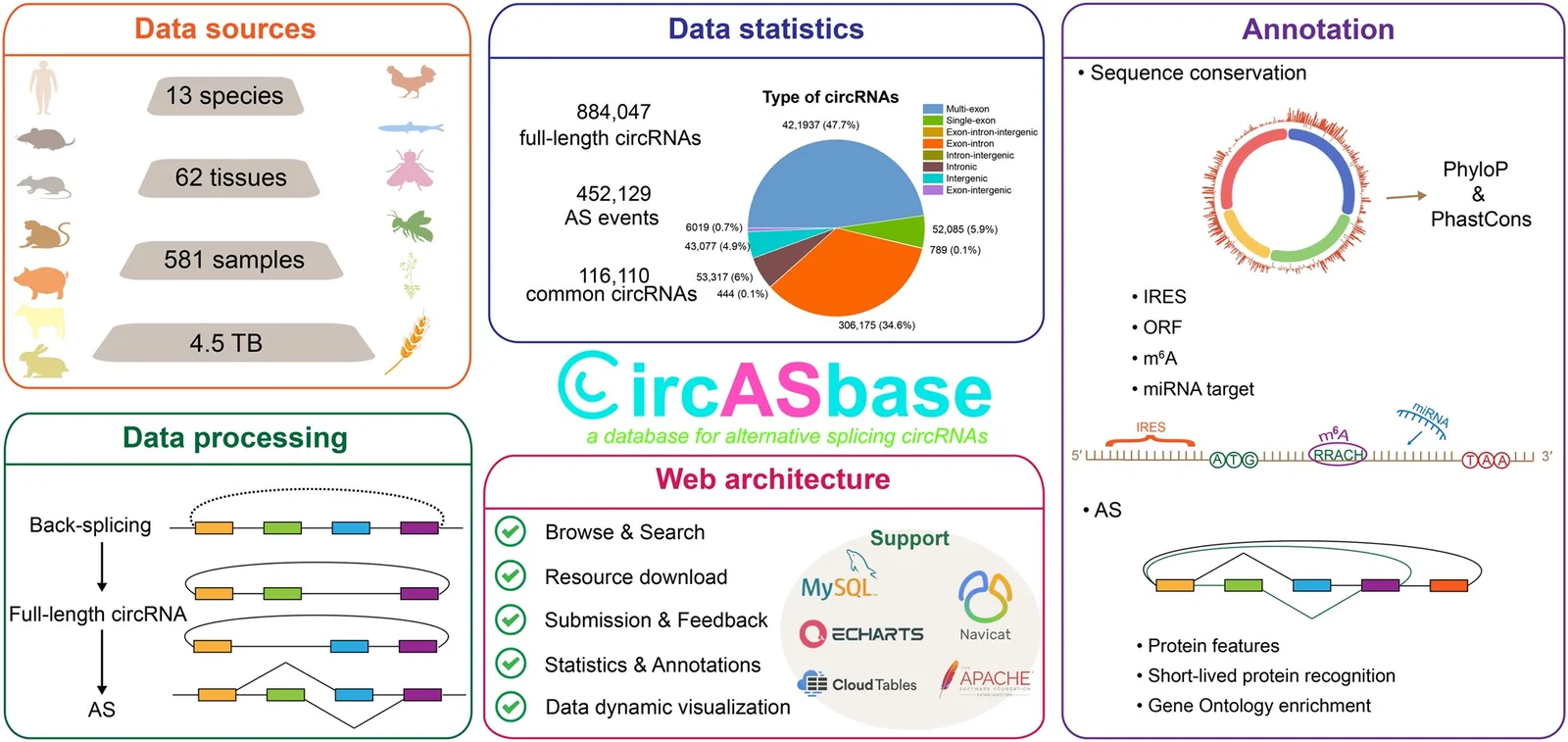
Database construction overview of circASbase Process of data collection, full-length circRNA prediction, AS event identification, circRNA annotation, and webpage construction. circRNA, circular RNA; TB, terabyte; IRES, internal ribosome entry site; ORF, open reading frame; m^6^A, *N*^6^-methyladenosine; miRNA, microRNA; AS, alternative splicing.

To develop a high-quality circRNA database, we systematically searched the Sequence Read Archive (SRA) database (https://www.ncbi.nlm.nih.gov/sra/) until January 21, 2022, using two key terms: “RNA-seq” and “RNase R”. Our final database is exclusively composed of RNA-seq data derived from samples treated with RNase R, an exoribonuclease that selectively degrades linear RNAs, thereby enriching for circRNAs [32,33]. A total of 581 samples were collected (Table S1), including 13 species (*Homo sapiens*,* Mus musculus*,* Rattus norvegicus*,* Macaca mulatta*,* Oryctolagus cuniculus*,* Bos taurus*,* Sus scrofa*,* Gallus gallus*,* Danio rerio*,* Apis mellifera*,* Drosophila melanogaster*,* Arabidopsis thaliana*, and* Oryza sativa*). The structure and content of circASbase are depicted in **Figure 1**. Briefly, circRNAs were identified using CIRI2 [34], with the exception of those obtained from publicly available projects. Full-length sequences were assembled with CircAST [31]. For circRNAs that could not be processed by CircAST, their complete sequences were assembled based on reference genomes and corresponding annotation files (Table S1). These full-length sequences were also annotated for components and internal structures. Potential AS events were detected using chromosomal coordinates and internal structures. In addition, functional elements such as internal IRESs, ORFs, m^6^A sites, miRNA targets, protein glycosylation sites, and phosphorylation sites on full-length circRNAs or their coding proteins were predicted. Sequence conservation was evaluated using PhastCons and PhyloP models [35,36]. Finally, we developed circASbase with dynamic visualization and database management features, offering search, browse, and download capabilities.

### Full-length assembly of circRNAs

We employed the BWA-MEM algorithm to align reads to the reference genome [37]. Subsequently, CIRI2 was used to identify BSJs, and CircAST reconstructed the internal structures of circRNAs [31,34]. This pipeline enabled us to obtain numerous high-confidence full-length circRNA sequences (File S1). Since CircAST is an exon annotation-based approach to constructing splicing graphs, it may lose some circRNAs composed of intronic or intergenic sequences. For BSJ sites that cannot be assembled by CircAST, we directly concatenated exons between the back-splice regions (Figure S1A). This may result in the inclusion of introns and intergenic regions in circRNAs, as BSJ sites are not always located in the exons.

Additionally, we collected full-length circRNA sequences from open-source projects or databases, comprising 286,696 for *H. sapiens* (Figure S1B), 6519 for *O. sativa* (including 466 validated), and 4663 for *A. thaliana* (including 137 validated) [29,38]. To eliminate redundancy, we used the CD-HIT-EST algorithm to remove circRNAs with over 95% sequence identity [39].

### AS events in circRNAs

We performed annotation of circRNA isoforms for each species in GTF format and established a pipeline to identify and classify AS events in circRNAs, based on the integration of our annotations with the reference genome annotation from the Ensembl database. Our strategy categorizes AS events into seven types: alternative 3′ splice site (A3SS), alternative 5′ splice site (A5SS), exon skipping (ES), exclusive exon skipping (EES), intron retention (IR), alternative 3′ back-splice site (A3BS), and alternative 5′ back-splice site (A5BS).

### Sequence conservation analysis

PhyloP evaluates only the current column of the alignment, while PhastCons also takes neighboring columns into account, making PhastCons more sensitive to detecting conserved regions and PhyloP more effective at defining them [35]. Using the chromosomal positions of circRNAs, we extracted sequence conservation scores (PhastCons and PhyloP) from the UCSC database with bwtool [36]. Since conservation data were available only for *H. sapiens*, *M. musculus*, *R. norvegicus*, and *G. gallus*, our conservation analysis focused on these species. circRNAs with an average PhastCons score > 0.6 and an average PhyloP score > 2 [40,41] were considered highly conserved.

### Large-scale functional annotation of circRNAs

We used IRESfinder and SRAMP to identify potential IRESs and m^6^A sites in circRNAs, respectively [42,43]. To determine the longest ORF in circRNAs, we first replicated and concatenated the circRNA sequence three times to simulate a circular reading frame. Then, we extracted all start and stop codons from the amplified sequence and selected the farthest pair as the ORF, which allows for overlapping ORFs in circRNAs that undergo looping. Approximately 60% of the ORFs of circRNAs cross the BSJ (Figure S3). Using SProtP [44], we predicted the half-lives of proteins encoded by ORFs in human circRNAs. For glycosylation site prediction, we used NetCGlyc for *C*-mannosylation, NetNGlyc for *N*-linked glycosylation, and NetOGlyc for *O*-GalNAc glycosylation [45–47]. We also employed GPS (v2.1) with a high-threshold model to predict phosphorylation sites [48]. We used TargetScan, miRanda, and RNAhybrid to identify potential miRNA binding sites in circRNAs [49–51], with mature miRNA sequences sourced from the miRBase database.

### Database construction and implementation

The core framework of circASbase utilizes Apache as the web server and MySQL as the database server. All data are managed within the MySQL database system. To enhance user experience, we developed the interface primarily using JavaScript, and implemented tables using DataTables jQuery plugin (v1.11.4). For data visualization, we employed ECharts (v5.2.1), a JavaScript library for creating interactive charts. The web services of circASbase have been tested on several popular browsers, including Google Chrome, Microsoft Edge, and Firefox.

## Discussion

**Figure 2:**
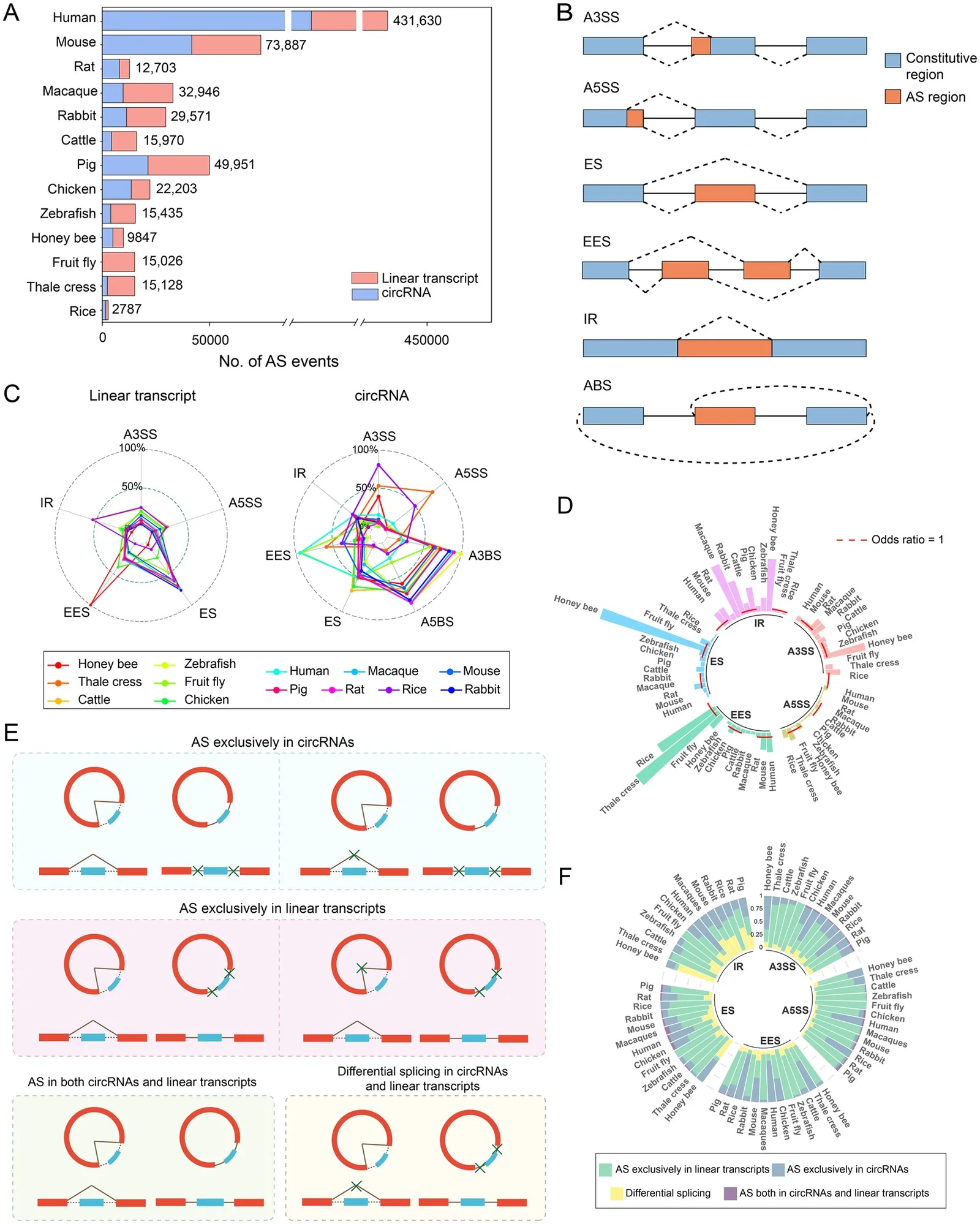
Statistics and analysis of AS events **A**. Statistics of AS events in circRNAs and linear transcripts of 13 species. **B**. Classification diagram of AS events. Blue boxes indicate constitutive regions shared among isoforms, whereas orange boxes indicate AS regions. **C**. Odds ratios of five AS types in circRNAs *vs.* linear transcripts. An odds ratio > 1 indicates preferential inclusion in circRNAs. **D**. Distribution of different types of AS events in linear transcripts and circRNAs from 13 species. **E**. Classification diagram of different AS events between circRNAs and linear transcripts. The classification principle is that when two splicing patterns occur in the same region of circRNAs, they are counted as AS events of circRNAs. The same applies to linear transcripts. The blue boxes represent regions where AS events occur, the red boxes represent regions without AS, and the cross symbol indicates that the corresponding splicing pattern does not exist. **F**. Statistics of different AS events in circRNAs and linear transcripts. AS events specific to circRNAs or linear transcripts account for the majority, whereas shared AS events remain rare. A3SS, alternative 3′ splice site; A5SS, alternative 5′ splice site; ES, exon skipping; EES, exclusive exon skipping; IR, intron retention; ABS, alternative back-splice; A3BS, alternative 3′ back-splice site; A5BS, alternative 5′ back-splice site.

**Figure 3:**
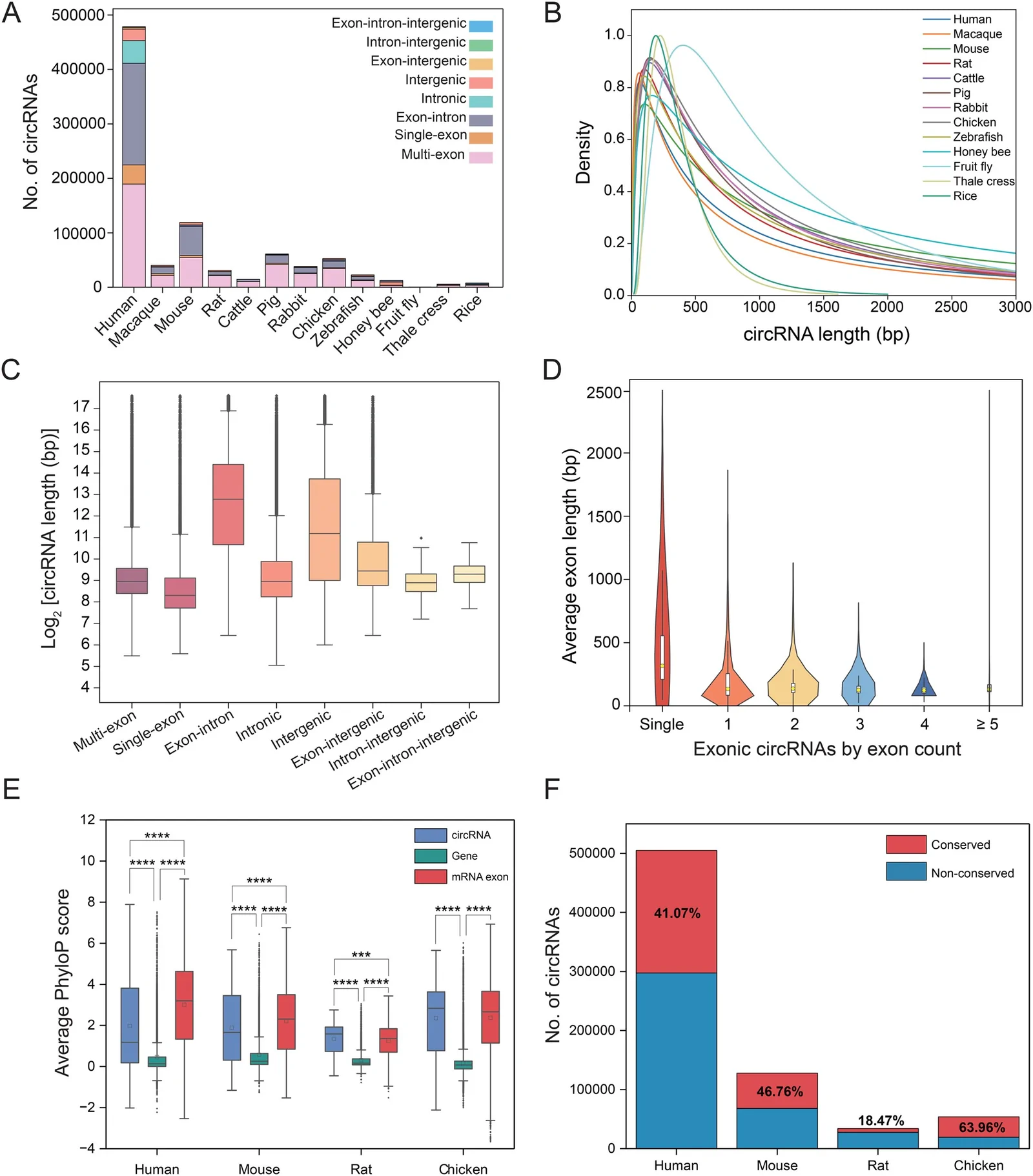
Structural characteristics and conservation of circRNAs **A**. Number of circRNAs per species and distribution of different types. **B**. Density distribution of circRNA length in each species. To exclude the effect of ultra-long circRNAs on the graphs, only data in the range of 0–3000 bp are shown. **C**. Box plot showing the length distribution of different types of circRNAs. **D**. Average length of exons in different types of circRNAs. “Single” denotes strictly defined single-exon circRNAs, which consist solely of sequences derived from a single exon with no other genomic elements. The labels “1”, “2”, “3”, “4”, and “≥ 5” denote other exonic circRNAs containing 1, 2, 3, 4, and ≥5 exons, respectively, including subtypes such as exon-intron circRNAs and multi-exon circRNAs. **E**. Average PhyloP scores of circRNAs, genes, and mRNA exons. *, *P* < 0.05; **, *P* < 0.01; ***, *P* < 0.001; ****, *P* < 0.0001 (Student’s *t*-test). **F**. Number of highly conserved circRNAs identified with average PhastCons score > 0.6 and average PhyloP score > 2.

**Figure 4:**
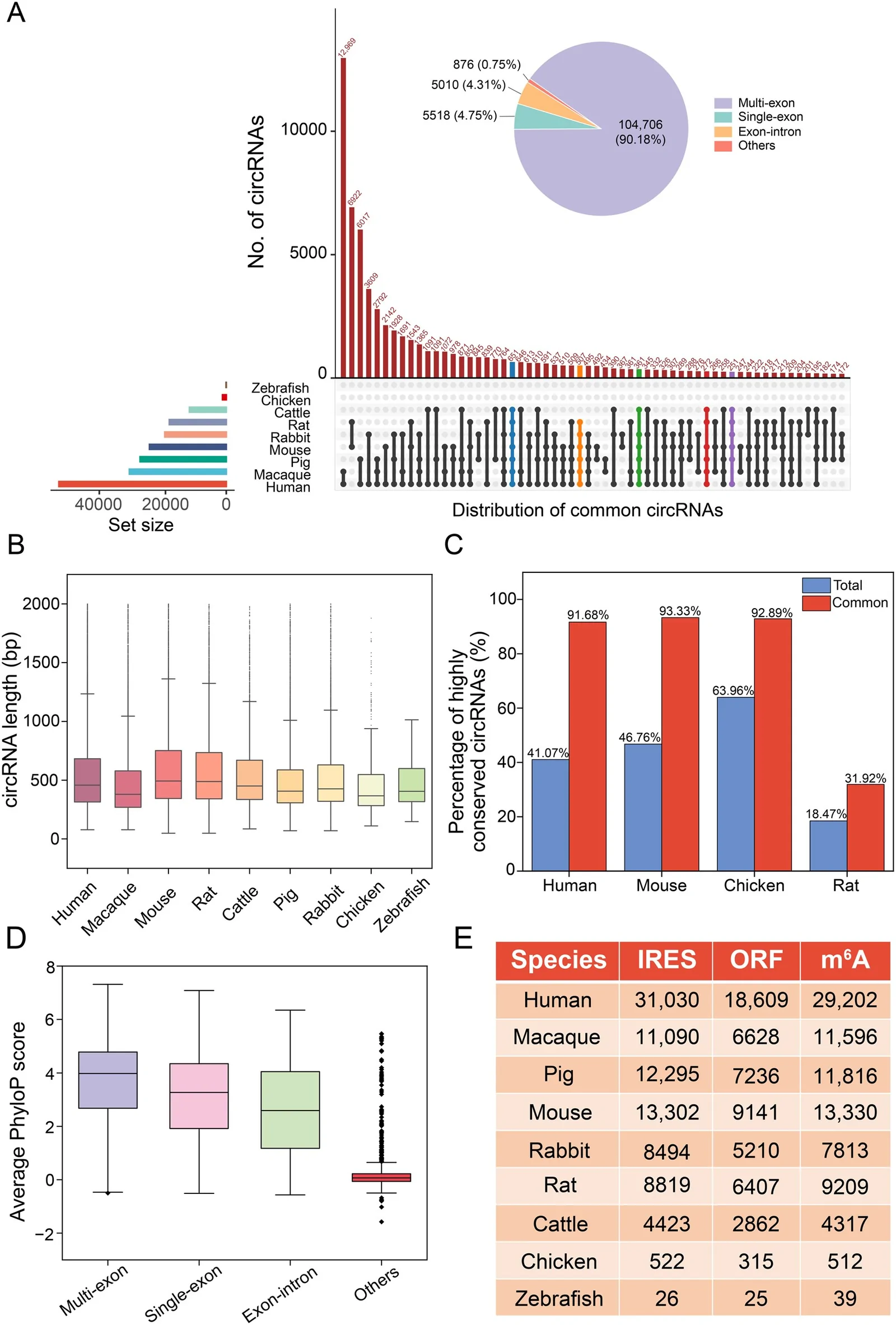
Characterization of common circRNAs in nine animals **A**. UpSetR plot showing the intersection patterns of circRNAs shared by at least two species. Horizontal bar graphs indicate the total number of common circRNAs identified in each species, and the connecting lines indicate different shared patterns among species; patterns shared in more than five species are labeled in color. Vertical bar graphs indicate the number of common circRNAs in the corresponding sharing mode. The pie chart depicts the classification of these common circRNAs, with multi-exon circRNAs being the predominant subtype. **B**. Length distribution of common circRNAs in each species. **C**. Percentage of highly conserved circRNAs in two groups (average PhyloP score > 2 and average PhastCons score > 0.6). **D**. Conservation assessment (PhyloP) of different types of common circRNAs. **E**. Statistics of common circRNAs containing potential coding elements in each species.

Here, we innovatively developed an interactive database that integrates AS and coding potential data for full-length circRNAs. This is the first comprehensive database to include full-length circRNAs from animals, insects, and plants. To construct this database, we collected 581 samples from 13 species, processed approximately 4.5 terabytes (TB) of data, and incorporated circRNA sequences from external sources. In total, we identified 884,047 full-length circRNAs, with 452,129 AS events detected (**Figures 2**A and **3**A). Additionally, through homology and sequence similarity analysis, we identified 116,110 conserved circRNAs shared by at least two species (**Figure 4**A).

### AS events in circRNAs

In this study, we comprehensively examined AS events in covalently closed circRNAs, which lack free 3′ or 5′ ends. A total of 452,129 AS events were identified across circRNAs from 13 species (Figure 2A). Of these, 329,269 events were observed in *H. sapiens*, whereas only 43 events were detected in *D. melanogaster*. It is important to note that the differences in the number of events may reflect variations in sample sizes rather than species-specific phenomena. A detailed summary of these results is shown in Table S2.

We further analyzed the distribution of AS events in circRNAs and found that EES was the most common event, accounting for 30.1% of all cases. This was followed by ES (20.1%), A5BS (14.4%), A3BS (14.3%), and IR (11.3%) (Table S2). Based on these AS events, we obtained 2,372,838 circRNA pairs (defined as pairs sharing one or more AS events) and assessed the effect of different splicing patterns on the functional elements of circRNAs. The results indicated that for most of the circRNA pairs, different splicing patterns resulted in alterations in ORFs, IRESs, and m^6^A sites (Figure S1D–F), which may affect the function of circRNAs [52,53].

### Comparison of AS between circRNAs and their corresponding linear transcripts

The identification of AS events in circRNAs allows for a comparative analysis of their distribution compared to their corresponding linear transcripts across multiple species (Table S3). Our study identified 452,129 AS events in circRNAs and 275,001 in their linear counterparts across 13 species (Figure 2A and B). Interestingly, in linear transcripts, ES is the predominant splicing event in most species, except in insects (*e*.*g.*, *A. mellifera* and *D. melanogaster*), where EES is most prevalent, and in plants, where IR is the dominant event (Figure 2C). The proportions of A3SS and A5SS events are similar across all species.

In contrast, circRNAs exhibit distinct AS patterns. A3BS and A5BS events are specific to circRNAs and are abundant across most species. To further explore the differences in AS events between circRNAs and linear transcripts, we calculated the odds ratios for the five types of AS events common to both RNA types (Figure 2D). Our analysis revealed a higher incidence of IR events in circRNAs, with most species displaying a similar distribution of AS events between circRNAs and linear transcripts. However, circRNAs exhibit a higher frequency of AS events in plants and insects compared to their linear transcripts. In summary, our findings highlight significant interspecies variation in the distribution of AS events between circRNAs and linear transcripts.

To further characterize the differences in AS between circRNAs and linear transcripts, we classified the events into four types based on full-length sequences: (1) AS events exclusively in circRNAs, (2) AS events exclusively in linear transcripts, (3) AS events in both circRNAs and linear transcripts, and (4) differential splicing patterns between circRNAs and linear transcripts (Figure 2E). The key criterion in this classification is whether the two splicing modes corresponding to an AS event occur in both circRNAs and their corresponding linear transcripts. Although the proportions of these types vary across the five event categories (A3SS, A5SS, ES, EES, and IR), 98% (616,816/629,298) of the events are not shared between circRNAs and linear transcripts (Figure 2F, Figure S2), with shared events being relatively rare. In addition, we assessed the conservation between circRNA-specific and linear transcript-specific fragments in AS events. We found that circRNA-specific splicing fragments were more conserved than linear transcript-specific splicing fragments (Figure S3A and B).

### Structural characteristics and conservation of circRNAs

Previous research has demonstrated that circRNAs vary widely in length, with exonic circRNAs ranging from 200 to 1000 bp and intergenic circRNAs typically exceeding 3000 bp [54,55]. We analyzed the length distribution of circRNAs across 13 species (Figure 3B) and found that in most animals, circRNAs are shorter than 3000 bp. The length distribution in other animal species resembles that of *H. sapiens*, although it is more dispersed. Notably, plant circRNAs are significantly shorter than those of animals, predominantly between 200 and 600 bp. In contrast, insect circRNAs are longer and more dispersed compared to those of other species (Figure 3B). These findings suggest substantial interspecies variation in circRNA lengths, particularly among animals, plants, and insects.

Given that length differences may be attributed to the internal structure of circRNAs, we categorized them into eight types: single-exon, multi-exon, exon-intron, intronic, intergenic, exon-intergenic, intron-intergenic, and exon-intron-intergenic. Approximately half of these circRNAs consist of multiple exons (multi-exon), and more than one-third of circRNAs contain exons and introns (exon-intron). Roughly 6% of circRNAs consist of a single exon (single-exon), and about 89% of circRNAs contain 1 to 5 exons. Conversely, around 11% of circRNAs are entirely intronic or intergenic, with this subset primarily observed in *H. sapiens* (Figure 3A). Furthermore, several circRNAs (exon-intergenic, intron-intergenic, and exon-intron-intergenic) cross the known boundaries of their host genes.

Most circRNAs fall within the range of 500–800 bp (Figure 3C), with IR significantly increasing their length. Notably, the length difference between single-exon circRNAs and other exonic circRNAs is smaller than expected (Figure 3C), suggesting that single-exon circRNAs may have longer exons. To better understand their differences, we calculated the average exon length and exon count for each circRNA type. The results revealed that exons in single-exon circRNAs are substantially longer than those in other exonic circRNA types (Figure 3D), corroborating previous findings [38,56,57].

Moreover, we examined the conservation of circRNAs. Based on PhyloP and PhastCons scores, circRNAs are slightly less conserved than mRNA exons, but more conserved than entire genes (Figure 3E, Figure S3C). We considered circRNAs with average PhyloP score > 2 and average PhastCons score > 0.6 as highly conserved, with approximately 40% of circRNAs being highly conserved (Figure 3F). Additionally, the regions near BSJ sites exhibit remarkable conservation (Figure S3D–G).

In addition, we compared circRNAs from four shared species in circASbase and circAtlas (v3.0), and 56.7% of BSJs (*H. sapiens*: 47.2%; *M. musculus*: 70.4%; *R. norvegicus*: 70.5%; *S. scrofa*: 87.0%) from circASbase were the same as those in circAtlas (Figure S4A). For circRNAs with the same BSJs, we compared their full-length sequences in the two databases and found 87,107 circRNAs (*H. sapiens*: 43,414; *M. musculus*: 19,554; *R. norvegicus*: 7873; *S. scrofa*: 16,266) that were completely identical (100% similarity) in both databases (Figure S4B).

### Identification of common circRNA isoforms derived from orthologous genes

Recent studies have suggested that circRNAs are closely associated with species identity [6,58–60]. Orthologous genes producing circRNAs generally possess more conserved splicing sites and exons than other genes [61]. To explore conserved circRNA isoforms and understand their genetic basis through evolutionary analysis, we conducted a sequence similarity analysis of circRNA isoforms generated by orthologous genes. A total of 116,110 eligible circRNAs were identified in at least two of the nine species (Figure S4C). In addition, 651 circRNAs were expressed in all seven mammalian species (Figure 4A). These findings indicate that shared circRNAs between species are a common phenomenon. Furthermore, species that are evolutionarily closer, such as *H. sapiens* and *M. mulatta*, or *M. musculus* and *R. norvegicus*, tend to share a higher number of circRNAs.

Similarly, we investigated structural characteristics of circRNAs common in different species and found that the proportion of multi-exon circRNAs was significantly higher than that of all circRNAs (Figure 4A). The circRNAs common in different species contain very few introns and intergenic regions, which leads to further compression of the length range (Figure 4B). The circRNAs common in different species were in a highly conserved state (average PhyloP score > 2 and average PhastCons score > 0.6), although the scores varied slightly (Figure 4C, Figure S4D and E). The existence of introns and intergenic regions can significantly reduce the conservation of circRNAs common in different species (Figure 4D, Figure S4F). Since circRNAs shared by two or more species are mostly conserved, they are likely to play important functional roles. Almost all these common circRNAs are annotated with ORFs, IRESs, and m^6^A sites (Figure 4E), which means they may have strong coding potential.

### Browse and search

**Figure 5:**
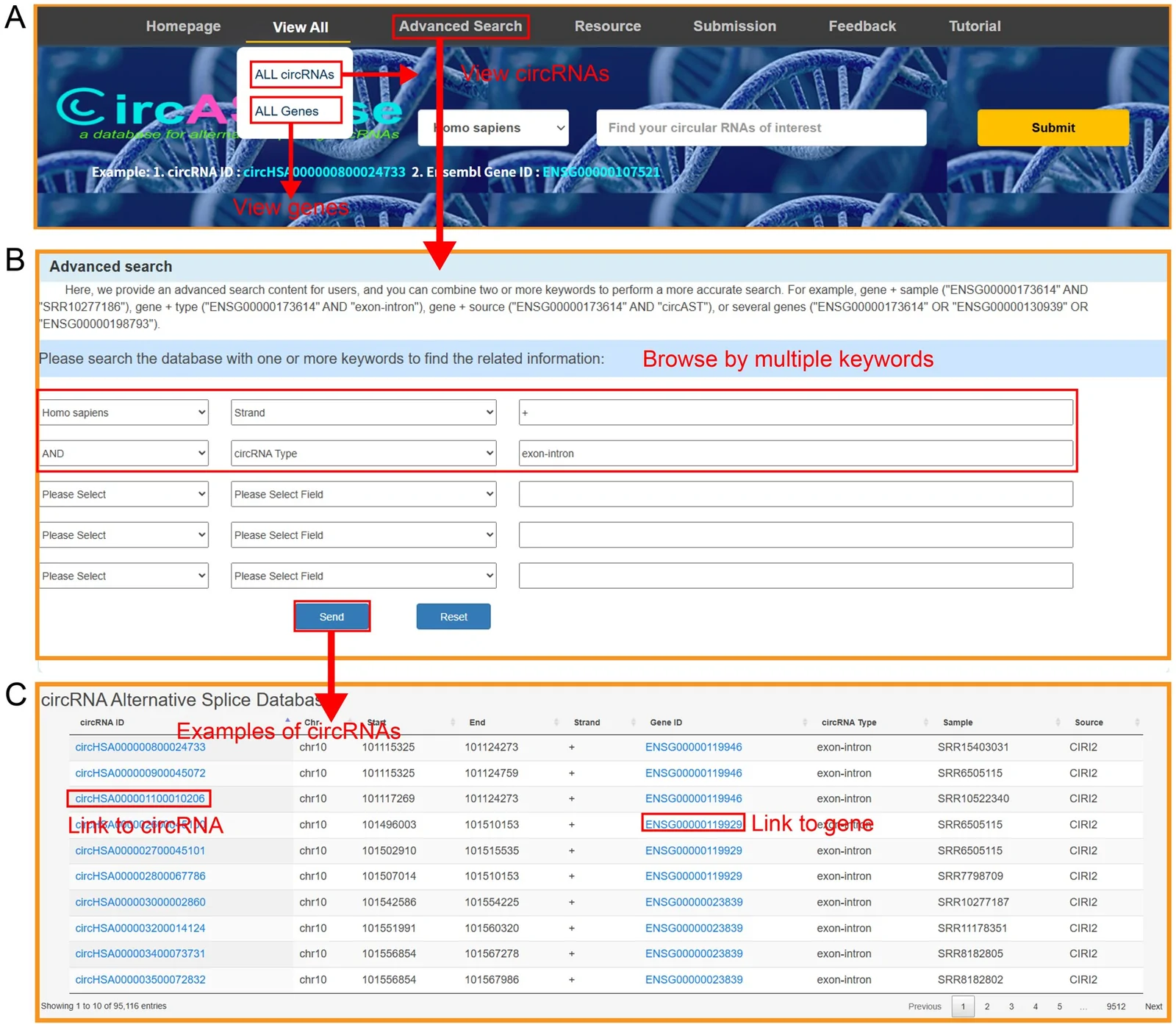
Screenshot of database browsing **A**. Navigation bar and homepage search section of the database, where circRNAs and genes can be separately browsed in the “View All” section or retrieved via the search box. **B**. Advanced search function, which allows searching by a combination of multiple keywords. **C**. Options for customized browsing of retrieved circRNAs and genes.

circASbase is a user-friendly database designed to support searching, browsing, and downloading of circRNA data. It utilizes web visualization technology to display AS events in circRNAs. Users can access the database in two ways: (1) by selecting a target species in the search box on the homepage and searching with specific keywords (*e.g.*, circRNA ID and gene ID) (**Figure 5**A); or (2) by using the advanced search page, where queries can be refined with keywords such as species, circRNA ID, gene ID, chromosome, strand, circRNA type, and sample name (Figure 5B). The query results are displayed in paginated tables, allowing users to further sort and filter the data by specified conditions (Figure 5C).

### Visualization

**Figure 6:**
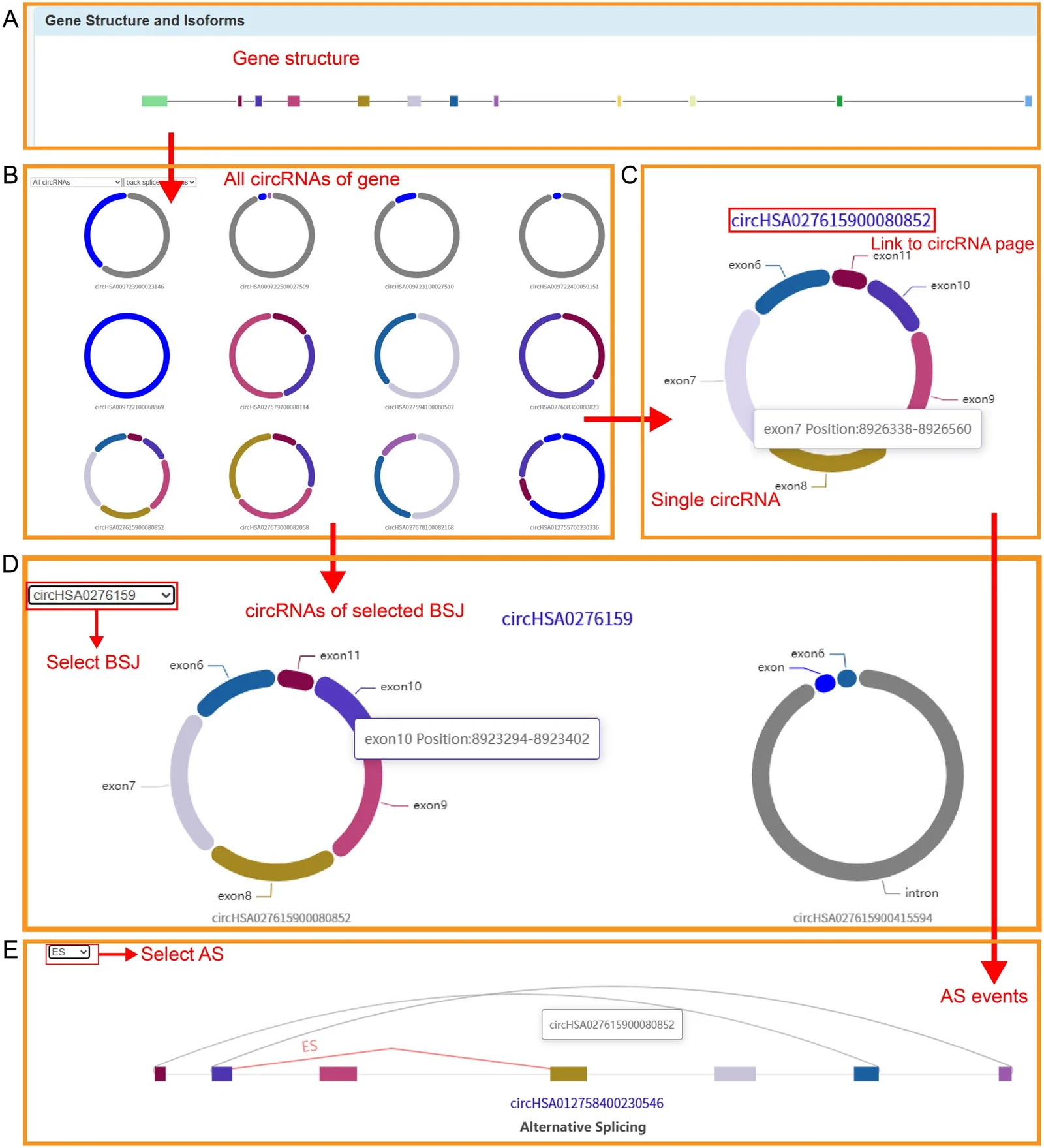
Page structure of AS events **A**. Display of the host gene structure. **B**. Charts of all circRNA isoforms of the queried gene. **C**. Schematic diagram of a single circRNA. The link can jump to the corresponding circRNA page. **D**. circRNAs with the same BSJ. **E**. Visualization of AS events associated with the selected circRNA. BSJ, back-splice junction.

**Figure 7:**
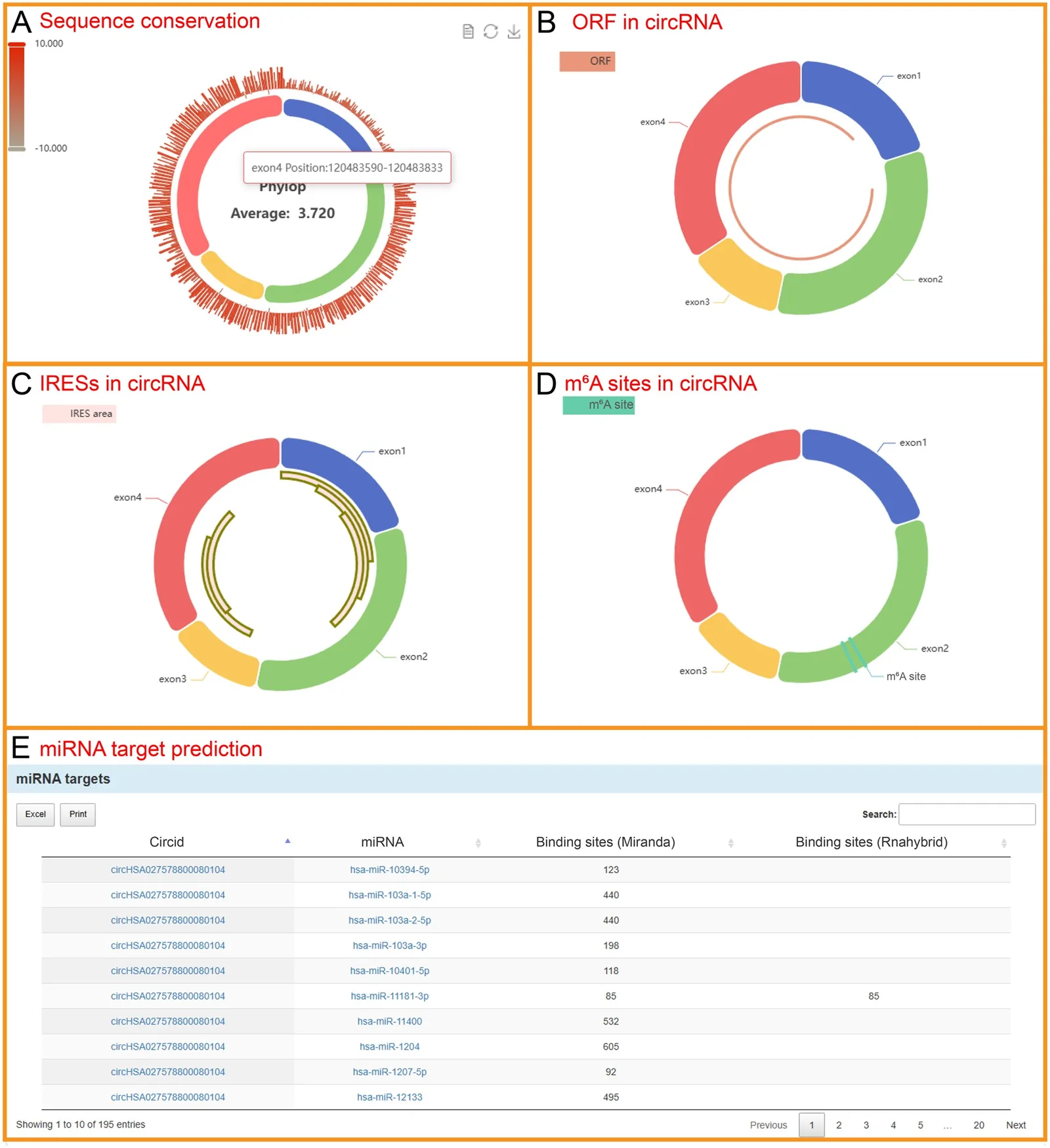
Overview of circRNA information page **A**. Schematic diagram of sequence conservation for the queried circRNA. **B**. Schematic diagram of the ORF in the queried circRNA. **C**. Schematic diagram of IRESs in the queried circRNA. **D**. Schematic diagram of m^6^A sites in the queried circRNA. **E**. Distribution table of miRNA targets in the queried circRNA.

circASbase provides intuitive visualization of AS events in circRNAs across 12 different species (*A. mellifera* excluded). The gene page displays the genomic structure in the top panel (**Figure 6**A), followed by diagrams of circRNA isoforms derived from the gene, where exons are color-coded to match the genomic structure (Figure 6B). A search function located in the upper left corner allows users to filter for specific circRNA isoforms by their unique IDs or back-splicing sites (Figure 6C and D). In addition to the genomic structure of circRNA isoforms, diagrams illustrating AS events between isoforms are provided. Users can selectively display specific splicing types to gain an intuitive understanding of these events (Figure 6E). Additionally, a dedicated detail page is available for each circRNA isoform, offering graphical representations of its sequence conservation, ORFs, IRESs, and m^6^A sites (**Figure 7**).

## Conclusion

In this study, we assembled 884,047 full-length circRNA isoforms with splicing annotations from 13 species. Our analysis revealed that the length distribution of circRNAs differs significantly at the kingdom level, with plant circRNAs being notably shorter than those of animals. Moreover, the exon length of single-exon circRNAs is significantly longer than that of other circRNA types. Additionally, we identified 116,110 orthologous circRNAs common in at least two species. We also observed that the AS events of circRNAs and linear transcripts differ markedly in both splicing forms and preferences, with few shared AS events.

In circASbase, we provide dynamic visualizations of AS events and annotate detailed information on circRNAs, including general information, full-length sequences, IRESs, m^6^A sites, ORFs, phosphorylation sites, protein glycosylation sites, conservation scores, and miRNA targets. Compared to existing circRNA databases [11,21,62], circASbase offers large-scale visualization of AS events at the full-length sequence level across 13 species from three kingdoms (animals, insects, and plants). In conclusion, circASbase serves as a valuable resource for understanding the mechanisms of AS in circRNAs and their functional roles.

## Supplementary Material

qzaf121_Supplementary_Data

## Data Availability

circASbase is freely accessible for non-commercial use at http://reprod.njmu.edu.cn/cgi-bin/circASbase/, and has been deposited in the Database Commons [63] at the National Genomics Data Center (NGDC), China National Center for Bioinformation (CNCB), which is publicly accessible at https://ngdc.cncb.ac.cn/databasecommons/database/id/9686.

## CRediT author statement

**Lingxiao Zou:** Methodology, Data curation, Formal analysis, Visualization, Writing – original draft. **Jian Zhao:** Project administration, Data curation. **Haojie Li: **Investigation, Visualization. **Chen Xu:** Investigation. **Yulan Wang:** Visualization. **Xuejiang Guo:** Conceptualization, Supervision, Writing – review & editing, Funding acquisition. **Xiaofeng Song:** Conceptualization, Supervision, Writing – review & editing, Funding acquisition. All authors have read and approved the final manuscript.
