# Supplementary material for: circASbase: A Comprehensive Database of Alternative Splicing Events in circRNAs
Source: Genomics Proteomics Bioinformatics. 2025 Dec 2;24(2):qzaf121. doi: 10.1093/gpbjnl/qzaf121 (PMC13563087; doi:10.1093/gpbjnl/qzaf121)
Supplement: Supplementary file 1 [file qzaf121_supplementary_data.zip › File S1.docx]

**File S1**

**Assembling workflow**

1. Build BWA index

**bwa** index genome.fa

2. Aligning to the reference genome

**bwa** mem -t 12 BWAIndex xxx_1.fastq xxx_2.fastq -o xxx_results.sam

3. Identify circRNAs

**perl CIRI2.pl** -I xxx_results.sam -O xxx_circList.txt -A genes.gtf -F genome.fa

4. Build Bowtie2 index

**bowtie2-build** genome.fa genome

5. Assemble circRNA transcripts

**tophat** -o results -p 12 -G genes.gtf Bowtie2Index xxx_1.fastq xxx_2.fastq

**samtools** sort -n -o accepted_hits.sorted.bam accepted_hits.bam

**samtools** view accepted_hits.sorted.bam > accepted_hits.sorted.sam

**python CircAST.py** -L 150 -T 5 -G genes.gtf -F accepted_hits.sorted.sam -J xxx_circList.txt

**python CircFullSeq.py** -F chromfafile_folder -I CircAST_result.txt -O circRNA.fa

**SeqCIRI.pl** -i CIRI2_result -f genome.gtf -g genome.fa -out circRNA.fa

**Remove redundant sequences**

**cd-hit-est** -i input.fa -o output.fa -c 0.95 -aL 0.9

**Identify AS events**

**AS_finder.pl** -g species.gtf -c circRNA_info.result -out AS_events
