# Supplementary material for: circASbase: A Comprehensive Database of Alternative Splicing Events in circRNAs
Source: Genomics Proteomics Bioinformatics. 2025 Dec 2;24(2):qzaf121. doi: 10.1093/gpbjnl/qzaf121 (PMC13563087; doi:10.1093/gpbjnl/qzaf121)
Supplement: Supplementary file 1 [file qzaf121_supplementary_data.zip › Supplementary material captions.docx]

Supplementary material

**File S1**

**Figure S1	Comprehensive analysis of circRNAs**

**A.** Proportion of full-length circRNAs assembled by CircAST or CIRI2 after removal of redundancy. **B.** Overlapped BSJs and full-length circRNAs of human. **C.** Statistics on ORFs of circRNAs crossing the BSJ sites in 13 species. **D.** Effect of AS events on ORFs in each circRNA pair. Darker color indicates ORF changed, lighter color indicates ORF not changed. **E.** Effect of AS events on IRESs in each circRNA pair. Darker color indicates IRESs changed, lighter color indicates IRESs not changed. **F.** Effect of AS events on m6A sites in each circRNA pair. Darker color indicates m6A sites changed, lighter color indicates m6A sites not changed.

**Figure S2	Percentage of AS events in circRNAs**

**Figure S3**	**The conservation score of circRNAs**

**A.** and **B.** The conservation score (Phylop and PhastCons) for circRNA-exclusive splicing fragments and mRNA-exclusive splicing fragments of four species. (Student's *t*-test, *, *P* < 0.05; **, *P* < 0.01; ***, *P* < 0.001; ****, *P* < 0.0001). **C.** Conservation assessment (PhastCons) of circRNAs, genes and mRNA exons. **D.**−**G.** Conservation assessment (Phylop and PhastCons) near the BSJ sites.

**Figure S4**	**Database comparison and common circRNAs**

**A.** Comparison of circASbase and circAtlas. Percentage of BSJs in circASbase that overlap with circAtlas. **B.** Comparison of full-length circRNAs from the same BSJ. A sizable number of full-length circRNAs (Similarity is 100%) can be found in both databases. **C.** Statistics of circRNAs common in two or more species. **D.**−**F.** Conservation assessment of circRNAs common in two or more species.

**Table S1	The statistics of full-length circRNA sequences and their corresponding samples**

**Table S2	Distribution of different types of circRNA splicing events in 13 species**

**Table S3	Distribution of different types of linear transcripts splicing events in 13 species**
