# Supplementary material for: circASbase: A Comprehensive Database of Alternative Splicing Events in circRNAs
Source: Genomics Proteomics Bioinformatics. 2025 Dec 2;24(2):qzaf121. doi: 10.1093/gpbjnl/qzaf121 (PMC13563087; doi:10.1093/gpbjnl/qzaf121)
Supplement: Supplementary file 1 [file qzaf121_supplementary_data.zip › Table S1.docx]

**Table S1	The statistics of full-length circRNA sequences and their corresponding samples**

| Species | No. of full-length sequences | No. of samples | No. of reference genome |
| --- | --- | --- | --- |
| Homo sapiens  Mus musculus  Rattus norvegicus  Macaca mulatta  Oryctolagus cuniculus  Bos taurus  Sus scrofa  Gallus gallus  Danio rerio  Apis mellifera  Drosophila melanogaster  Arabidopsis thaliana  Oryza sativa | 478,460  118,728  31,085  40,131  38,232  14,830  61,432  52,593  22,669  12,340  296  5503  7748 | 299  81  9  61  9  8  6  50  17  39  2  Collected  Collected | hg19  mm10  rn6  rheMac10  oryCun2  bosTau9  susScr11  galGal6  danRer11  apiMel2  dm6  TAIR10  IRGSP-1.0 |
