# Supplementary material for: circASbase: A Comprehensive Database of Alternative Splicing Events in circRNAs
Source: Genomics Proteomics Bioinformatics. 2025 Dec 2;24(2):qzaf121. doi: 10.1093/gpbjnl/qzaf121 (PMC13563087; doi:10.1093/gpbjnl/qzaf121)
Supplement: Supplementary file 1 [file qzaf121_supplementary_data.zip › Table S2.docx]

**Table S2	Distribution of different types of circRNA splicing events in 13 species**

| **Species** | **#A3SS** | **#A5SS** | **#A3BS** | **#A5BS** | **#ES** | **#EES** | **#IR** | **Total** |
| --- | --- | --- | --- | --- | --- | --- | --- | --- |
| H. sapiens | 20,360 | 17,301 | 29,079 | 29,763 | 67,798 | 124,940 | 40,028 | 329,269 |
| M. musculus | 1582 | 370 | 10,819 | 11,049 | 7756 | 5977 | 4149 | 41,702 |
| R. norvegicus | 150 | 26 | 2902 | 2819 | 1523 | 380 | 200 | 8000 |
| M. mulatta | 208 | 35 | 3405 | 3407 | 1241 | 323 | 1100 | 9719 |
| O. cuniculus | 260 | 43 | 3807 | 3807 | 1874 | 449 | 1087 | 11,327 |
| B. taurus | 79 | 21 | 1194 | 1158 | 1213 | 349 | 316 | 4330 |
| S. scrofa | 557 | 177 | 6168 | 6367 | 4284 | 1399 | 2365 | 21,317 |
| G. gallus | 275 | 66 | 3947 | 3883 | 3464 | 1150 | 715 | 13,500 |
| D. rerio | 158 | 26 | 1603 | 1395 | 455 | 62 | 287 | 3986 |
| A. mellifera | 781 | 78 | 1241 | 1188 | 1035 | 310 | 307 | 4940 |
| D. melanogaster | 0 | 1 | 10 | 5 | 9 | 16 | 2 | 43 |
| A. thaliana | 509 | 755 | 154 | 107 | 56 | 562 | 223 | 2366 |
| O. sativa | 530 | 328 | 176 | 106 | 25 | 253 | 212 | 1630 |
