# Supplementary material for: circASbase: A Comprehensive Database of Alternative Splicing Events in circRNAs
Source: Genomics Proteomics Bioinformatics. 2025 Dec 2;24(2):qzaf121. doi: 10.1093/gpbjnl/qzaf121 (PMC13563087; doi:10.1093/gpbjnl/qzaf121)
Supplement: Supplementary file 1 [file qzaf121_supplementary_data.zip › Table S3.docx]

**Table S3	Distribution of different types of linear transcripts splicing events in 13 species**

| **Species** | **#A3SS** | **#A5SS** | **#ES** | **#EES** | **#IR** | **Total** |
| --- | --- | --- | --- | --- | --- | --- |
| H. sapiens | 9609 | 9398 | 55,290 | 21,649 | 6415 | 102,361 |
| M. musculus | 3989 | 4014 | 16,985 | 4331 | 2866 | 32,185 |
| R. norvegicus | 401 | 362 | 2438 | 1080 | 422 | 4703 |
| M. mulatta | 732 | 960 | 14621 | 5491 | 1423 | 23,227 |
| O. cuniculus | 559 | 561 | 11,998 | 3926 | 1200 | 18,244 |
| B. taurus | 572 | 648 | 6433 | 2838 | 1149 | 11,640 |
| S. scrofa | 1376 | 1742 | 17,240 | 5941 | 2335 | 28,634 |
| G. gallus | 381 | 434 | 5468 | 1260 | 1163 | 8706 |
| D. rerio | 1707 | 1730 | 4734 | 1490 | 1788 | 11,449 |
| A. mellifera | 298 | 150 | 165 | 4216 | 78 | 4907 |
| D. melanogaster | 2627 | 2474 | 3151 | 5118 | 1656 | 15,026 |
| A. thaliana | 2667 | 2736 | 1182 | 264 | 5913 | 12,762 |
| O. sativa | 249 | 227 | 114 | 23 | 544 | 1157 |
